# Supplementary material for: Jahn-Teller distortion driven magnetic polarons in magnetite
Source: Nat Commun. 2017 Jun 29;8:15929. doi: 10.1038/ncomms15929 (PMC5493765; doi:10.1038/ncomms15929)
Supplement: Supplementary Information [file ncomms15929-s1.pdf]

Type of file: PDF
Size of file: 0 KB
Title of file for HTML: Supplementary Information
Description: Supplementary Figures, Supplementary Notes and Supplementary References

Type of file: PDF
Size of file: 0 KB
Title of file for HTML: Peer Review File
Description:

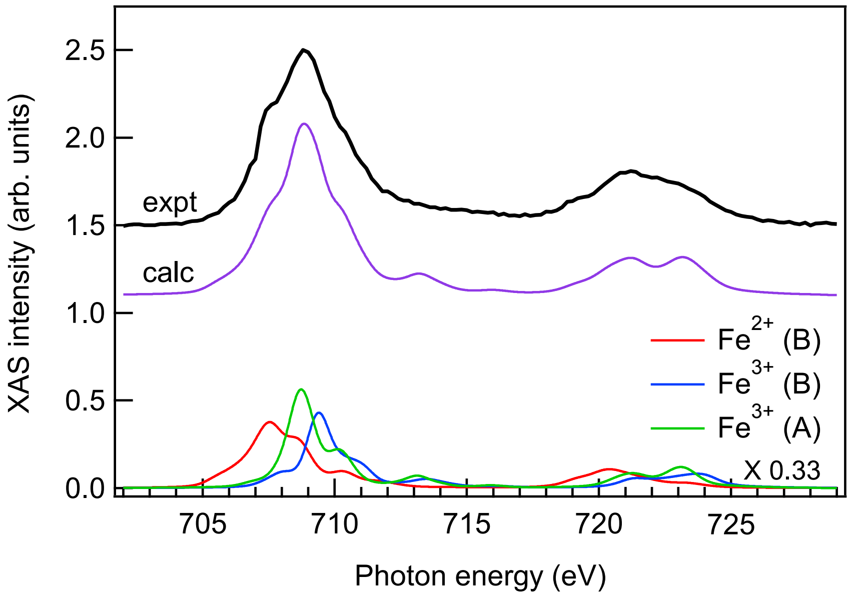

Supplementary Figure 1: Calculated and experimental XAS spectra of Fe_3O_4. The values of crystal field 10Dq are 1.13 eV for both octahedral Fe^{2+} and Fe^{3+}, and -0.6 eV for tetrahedral Fe^{3+}. A tetragonal distortion field $\Delta_{t_{2g}} = -24$ meV is applied on octahedral Fe^{2+}. The calculated XAS spectra is a combination of the three spectra with a relative weight of Fe^{3+}(A):Fe^{3+}(B):Fe^{2+}(B) $= 1 : 0.8 : 1.2$.

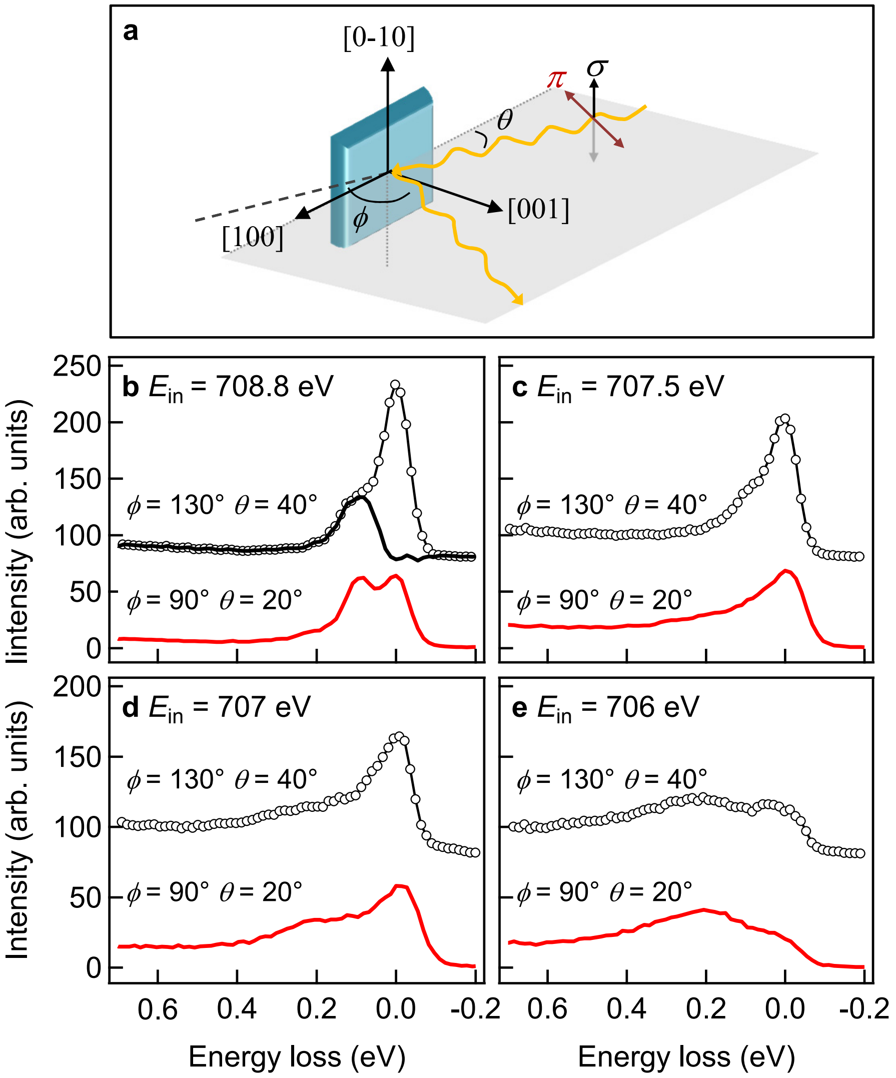

Supplementary Figure 2: RIXS spectra taken with different scattering geometries. (**a**) Illustration of the scattering geometry of RIXS measurements. (**b**)-(**e**) Fe L_3-edge RIXS spectra measured with different incident X-ray energies and scattering geometries. For incident photon energy E_{in} set to 706 eV, the scattering conditions ($\phi = 130°$, $\theta = 40°$) and ($\phi = 90°$, $\theta = 20°$) correspond to momentum transfer $\mathbf{q} = (-0.366, 0, 0.785)2\pi/a$ and $(-0.286, 0, 0.613)2\pi/a$, respectively. The RIXS spectra shown here were not corrected for self-absorption.

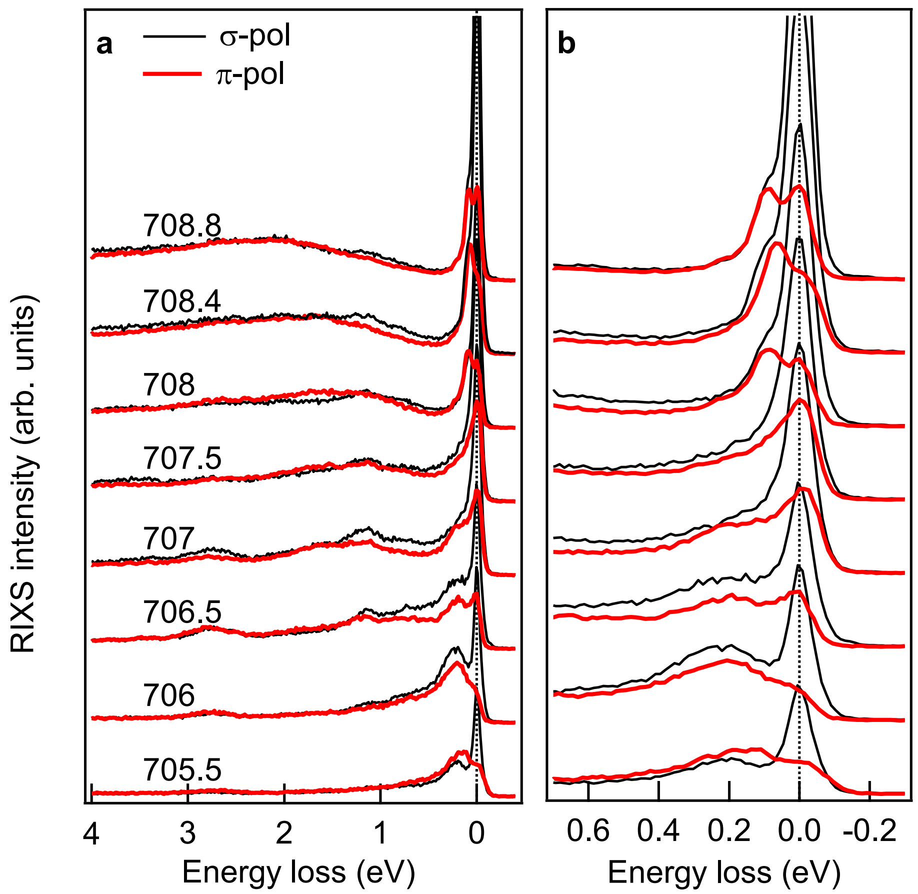

Supplementary Figure 3: **RIXS spectra taken with selected incident photon energies.** (**a**) RIXS spectra taken with π (thick red line) and σ (thin black line) polarised incident X-rays, respectively. (**b**) The magnified plot of energy loss below 0.7 eV. The scattering condition is $\phi = 90^\circ$ and $\theta = 20^\circ$ at temperature $T = 80$ K. All spectra were plotted without correction for self-absorption.

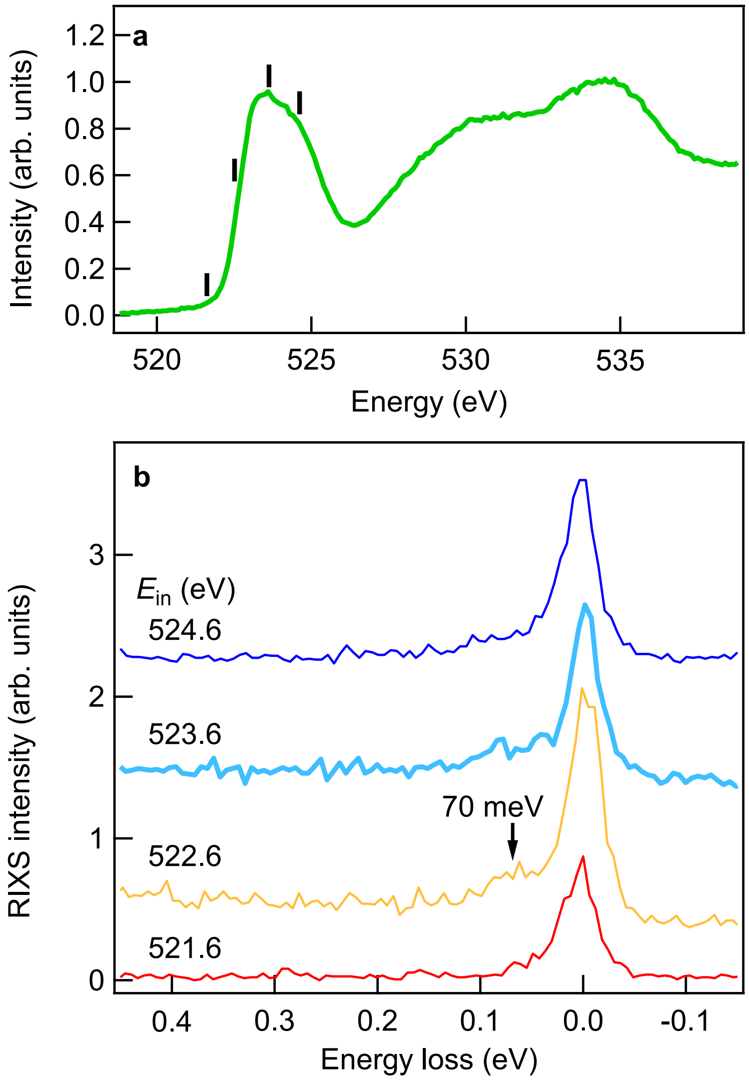

Supplementary Figure 4: **RIXS measurements of Fe_3O_4 at O** K**-edge.** (**a**) XAS spectrum taken at room temperature. Vertical bars indicate energies of incident X-ray: 521.6, 522.6, 523.6 and 524.6 eV. (**b**) RIXS spectra measured at selected incident photon energies. The scattering angle ϕ is 130° and incident angle θ is 20°. All spectra were recorded at 300 K.

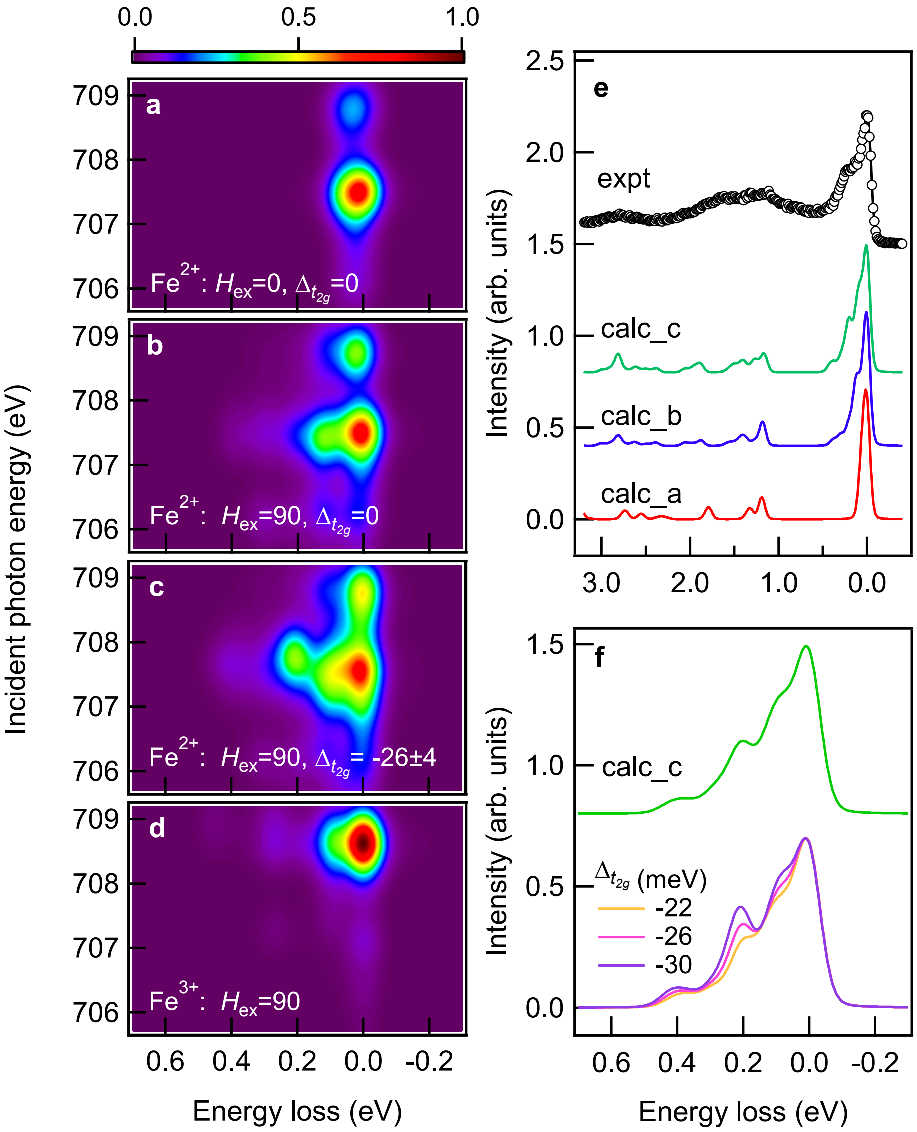

Supplementary Figure 5: **Calculated RIXS of Fe^{2+} and Fe^{3+}.** (**a**) & (**b**) Calculated RIXS intensity maps of Fe^{2+} with $H_{\text{ex}} = 0$, $\Delta_{t_{2g}} = 0$, and $H_{\text{ex}} = 90$ meV, $\Delta_{t_{2g}} = 0$, respectively. (**c**) Calculated RIXS intensity maps of Fe^{2+} with $H_{\text{ex}} = 90$ meV, $\Delta_{t_{2g}} = -26 \pm 4$ meV, and (**d**) Fe^{3+} with $H_{\text{ex}} = 90$ meV, $\Delta_{t_{2g}} = 0$. The core-hole lifetime width is set to 200 meV and the final-state lifetime width is set to 10 meV. These intensity maps present the average RIXS intensity for the magnetic easy axis along the [100], [010] and [001] directions and are plotted after Gaussian broadening of width 500 meV and 80 meV for the incident photon energy and the energy loss, respectively. (**e**) Comparison of measured (expt) and calculated (calc) RIXS spectra. Open circles are measurements with incident X-rays of 707 eV; solid lines calc_a, calc_b and calc_c are the corresponding RIXS spectra extracted from panels (**a**), (**b**) and (**c**) with the incident X-ray of 707.5 eV. (**f**) Calculated RIXS spectra using $H_{\text{ex}} = 90$ meV, and three different distortions in comparison with that shown in (**c**) with incident X-ray of 707.5 eV.

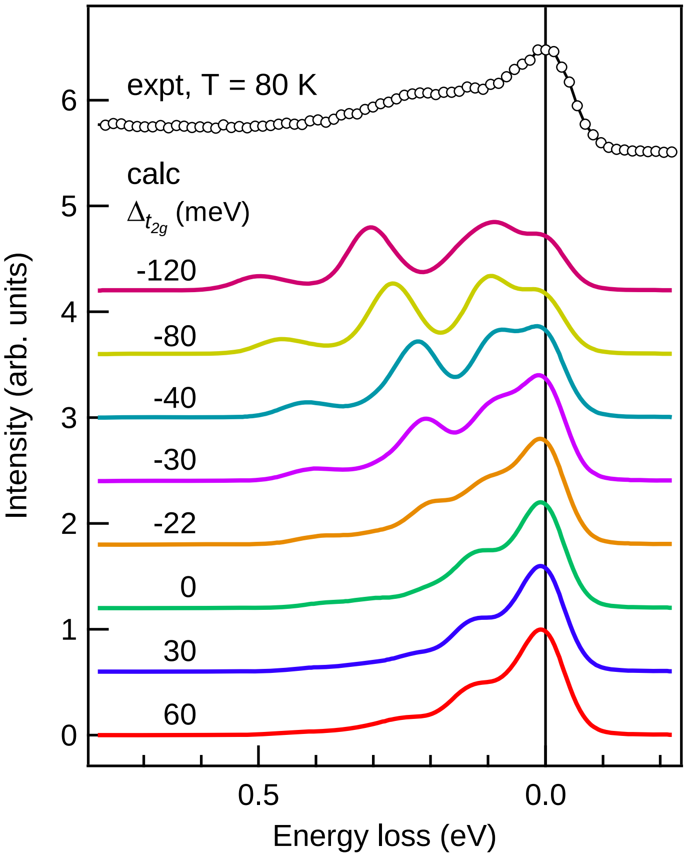

Supplementary Figure 6: **Calculated RIXS of Fe^{2+} of varied tetragonal distortion field.** The measured RIXS of Fe^{2+} (black) was recorded with the incident photon energy set at 707 eV. Calculated RIXS spectra with different tetragonal distortion field $\Delta_{t_{2g}}$ are plotted in color.

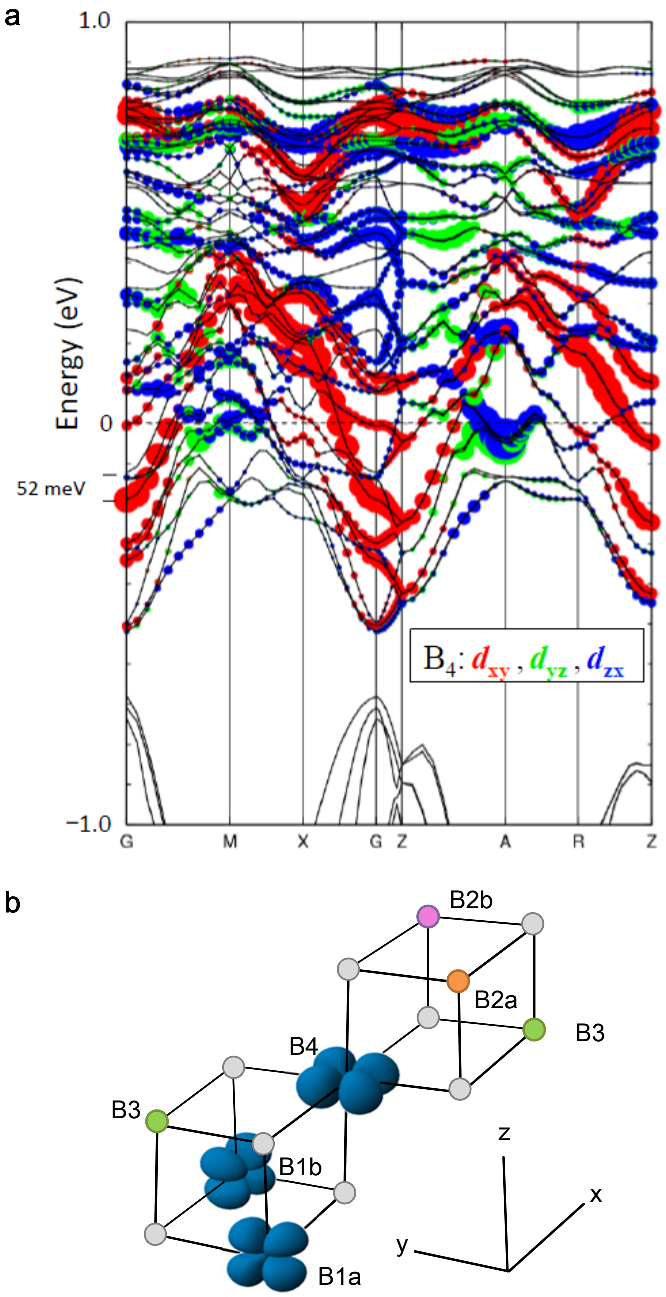

Supplementary Figure 7: Band structure of magnetite near the Fermi level. (**a**) Calculated minority-spin band structure of magnetite near the Fermi level. The calculations were in the GGA scheme under the low-T monoclinic $P2/c$ crystal structure. The minority-spin t_{2g} bands are highlighted for d_{xy} (red), d_{yz} (green) and d_{zx} (blue) orbitals of the B_4 site with circles of various sizes to indicate weights of the density of states. The splitting of 52 meV between the d_{xy} and $d_{yz/zx}$ bands at the Γ point due to the tetragonal distortion is indicated. (**b**) The corner-sharing B-site Fe tetrahedra of Fe_3O_4 with notations showing inequivalent B sites.

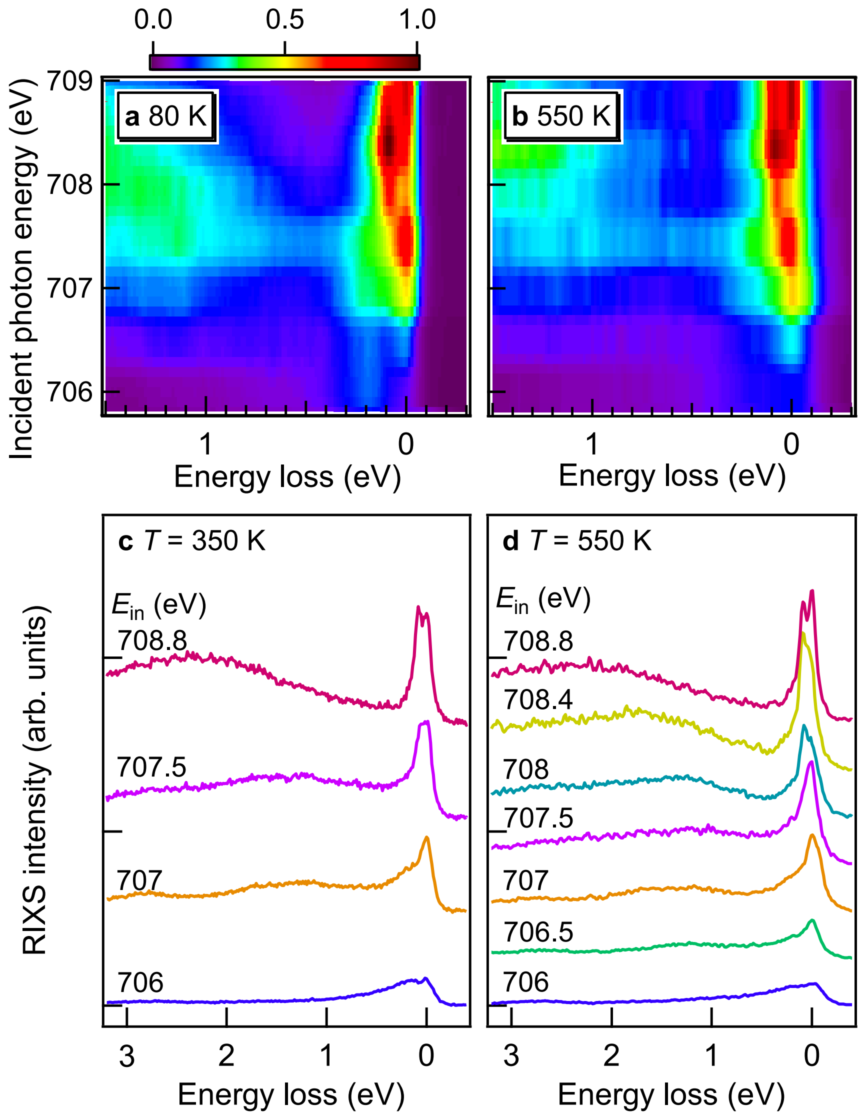

Supplementary Figure 8: **RIXS measurements of Fe_3O_4 at high temperatures.** (**a**) & (**b**) RIXS intensity maps after correction for self-absorption in the plane of incident photon energy vs. energy loss at 80 K and 550 K. (**c**) & (**d**) RIXS spectra plotted in terms of energy loss at 350 K and 550 K for selected incident X-ray energies.

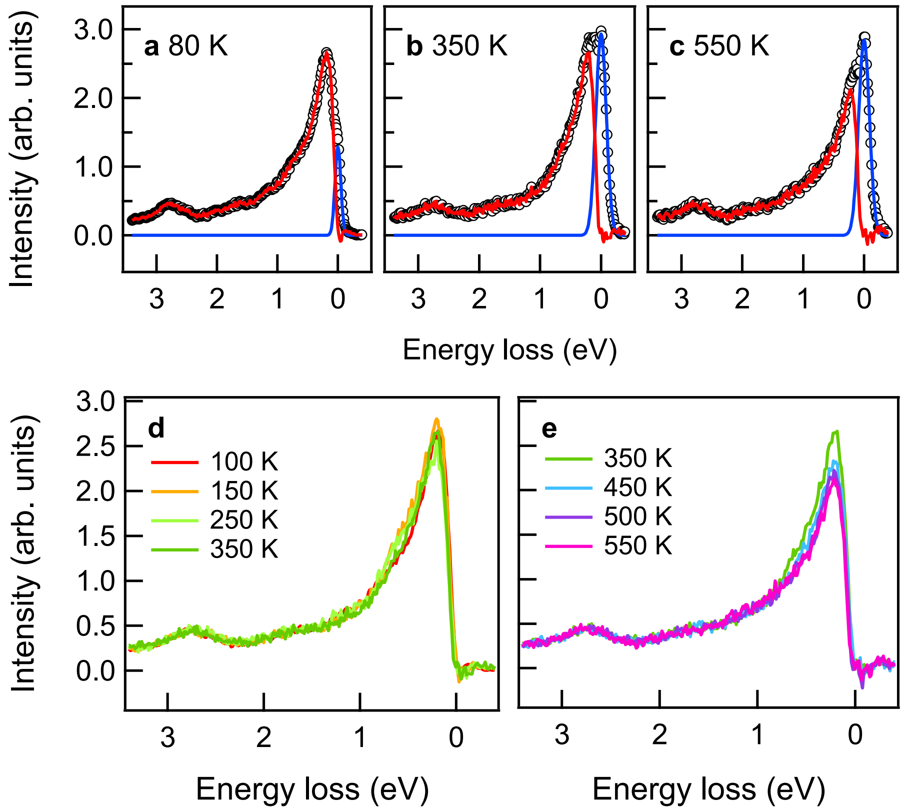

Supplementary Figure 9: **Comparison of RIXS spectra measured at selected temperatures.** (**a**), (**b**) & (**c**) RIXS spectra recorded at 80 K, 350 K, and 550 K. Open circles and blue solid lines show measured spectra and the elastic components, respectively. Red solid lines are spectra after the subtraction of the elastic component. (**d**) & (**e**) RIXS spectra after the subtraction of the elastic component and normalisation to the intensity of the 2.8 eV *dd* excitation feature at selected temperatures between 90 K and 550 K. All spectra were recorded with the incident X-ray energy set to 706 eV. The RIXS data comprise an average of four runs of experimental results.

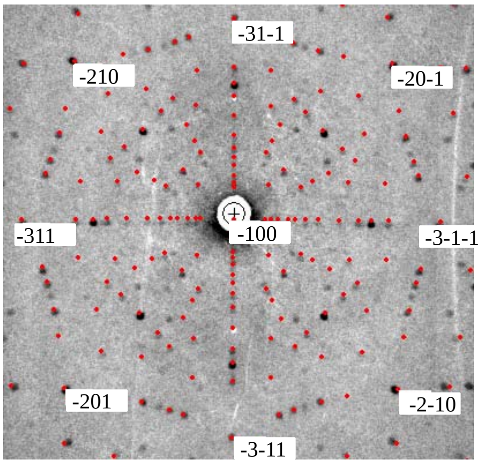

Supplementary Figure 10: **Laue back reflection pattern of Fe_3O_4 crystal.** Black features are the original Laue pattern. The red dots superimposed on the measured image are the corresponding orientation index. The software OrientExpress (https://www.ill.eu/instruments-support/computing-for-science/cs-software/all-software /orientexpress/) was used for orienting the crystal.

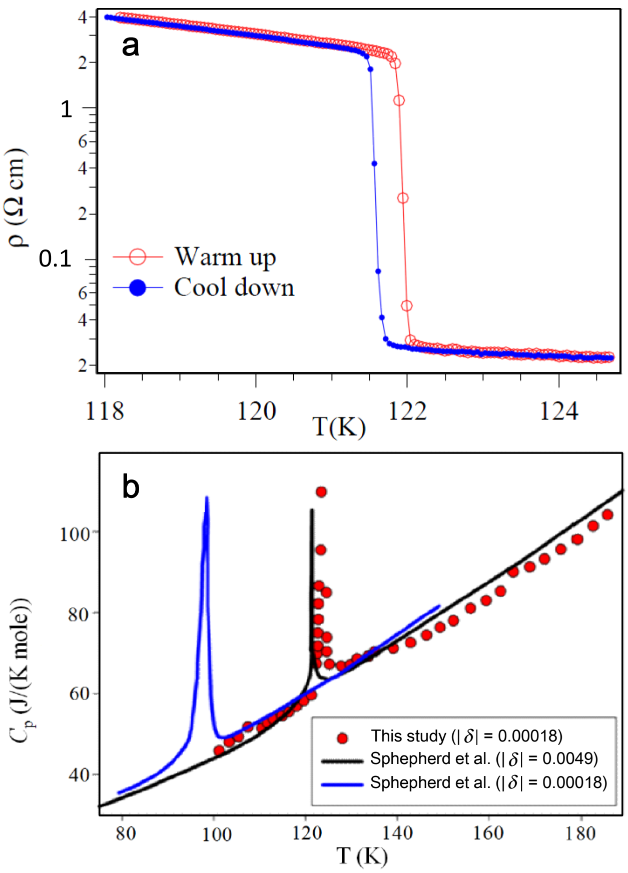

Supplementary Figure 11: **Resistivity and heat capacity measurements.**(**a**) Resistivity of single-crystal magnetite used in this study. (**b**) Heat capacity of single-crystal magnetites with different chemical stoichiometries.

Supplementary Note 1
RIXS measurements

Using the AGM-AGS spectrometer at beamline 05A1 of the National Synchrotron Radiation Research Center (NSRRC), Taiwan[1], we measured RIXS of Fe_3O_4 excited at selected incident photon energies and polarisations. The polarisation of incident X-ray was in the scattering plane or perpendicular to the scattering plane, i.e., π and σ polarisations, respectively. Supplementary Fig. 2(a) illustrates the scattering geometry in which the scattering angle, i.e. the angle between the incident and the scattered X-rays, is denoted as ϕ, and the incident angle from the ab plane is θ. The photon energy was set to specific energies about the L_3-edge ($2p_{3/2} \rightarrow 3d$) x-ray absorption of Fe to distinguish dominantly Fe^{2+} and Fe^{3+} derived dd excitations.

Supplementary Figures 2(b)-(e) show RIXS spectra which were recorded with a total energy resolution $\sim$ 80 meV. The polarisation of the incident X-ray was within the scattering plane (the ac plane), i.e. the π polarisation. To examine if the 200-meV excitation depends on the momentum transfer $\mathbf{q}$, we checked RIXS with two scattering geometries : $\phi = 130°, \theta = 40°$ and $\phi = 90°, \theta = 20°$. The incident X-ray energy was set to 706, 707, 707.5 and 708.8 eV. These data show that both 90-meV excitation and 200-meV spin-orbital excitations is momentum independent within the present energy and momentum resolutions.

In addition to momentum-dependent measurements, we also performed polarisation-dependent RIXS measurements at selected incident photon energies as plotted in Supplementary Fig. 3 in which the relative intensities between RIXS spectra of π and σ polarisations are normalised to background spectral intensities above an energy loss of 3 eV.

Supplementary Note 2
Crystal-Field Multiplet RIXS Calculations

We undertook crystal-field multiplet RIXS calculations of Fe^{2+} using CTM4RIXS[2] and MISSING (Dallera, C. and Gusmeroli, R. http://www.esrf.eu/computing/scientic/MISSING/) with the scattering angle 90° and the magnetisation axis perpendicular to the scattering plane or in the scattering plane with angles 20° or 70° to the incident beam. The polarisation of incident X-rays was selected to be π polarised. Since the polarisation of scattered X-rays was not analysed in the measurements, we summed calculated RIXS spectra of scattered X-rays with σ and π polarisations.

Traditionally, a spectrum of L_3-edge XAS is broadened with a Lorentzian width 0.4 eV for lifetime broadening and a Gaussian width to simulate the experimental broadening, but the Lorentzian broadening also takes into account broadening effects due to dispersion or band effects, vibrations and charge transfer. However, in principle, these effects

have a Gaussian lineshape and not Lorentzian, but this never posed a problem for XAS simulations given the small differences when the experiments are done with high energy resolution. But for RIXS experiments, the difference between Gaussian and Lorentzian is important because of the interference effects in the Kramers-Heisenberg equation. We found that lifetime Lorentzian broadening 0.2 eV for the intermediate states give results much better than 0.4 eV for the RIXS calculations. As discussed in the main text and in the following, the comparison shows that RIXS spectra can be nicely simulated with inclusion of an effective exchange field of 90 meV to account for the interatomic spin interactions among $3d$ electrons and a polaronic Jahn-Teller distortion.

The calculated spectra are obtained as an average of the spectra calculated for magnetic domains with the easy axis along the [100], [010] and [001] directions. The crystal field parameter $10Dq$ was set to 1.13 eV and the Slater integrals were reduced to 79% of their atomic values for accurately reproducing the dd excitation energies. With only the spin-orbit coupling strength $\zeta_{3d} = 52$ meV included, there exists low-energy excitation at 64 meV, but the 200 meV is not reproduced (Supplementary Fig. 5(a)). If an effective molecular field $H_{\mathrm{ex}} = 90$ meV is included without the tetragonal distortion, these excitations are split further with the excitation energy centroid at 132 meV, but still the 200 meV feature is not obtained (Supplementary Fig. 5(b)). We, therefore, need to either increase the effective molecular field to nearly 200 meV, or include the effect of the tetragonal distortion of FeO_6 octahedra. It is, however, unreasonable to use an molecular field much larger than the spin wave energy or the molecular field of Fe^{3+}, 90 meV. Hence, we included a tetragonal distortion for calculating the RIXS spectrum of Fe^{2+} in magnetite.

We performed a series of RIXS calculations for Fe^{2+} with $H_{\mathrm{ex}} = 90$ meV and as a function of tetragonal distortions as shown in Supplementary Fig. 6. A positive $\Delta_{t_{2g}}$, i.e. an elongated distortion along the local Jahn-Teller axis and contracted Fe-O bonds in the xy plane, does not yield correct energies of the excitations. For a small tetragonal distortion with -21 meV $< \Delta_{t_{2g}} < 0$, the effective exchange field dominates the low-energy excitations and results in an energy centroid ~ 120 meV. If the $\Delta_{t_{2g}}$ strength of the tetragonal compression is beyond -21 meV, the excitation profile is broadened dramatically with two major features; their excitation energies and the separation between them gradually increase with the increase of the $\Delta_{t_{2g}}$ strength. We found that $H_{\mathrm{ex}} = 90$ meV and $\Delta_{t_{2g}}$ about -24 meV most satisfactorily explain the measured RIXS spectra resulting from Fe^2+ states. The negative value of $\Delta_{t_{2g}}$ signifies that the energy of d_{xy} is lower than that of d_{yz}/d_{zx}.

With the parameters obtained from RIXS measurements and calculations, we verified if these parameters are consistent with the XAS spectrum. Supplementary Figure 1 presents a comparison between measured XAS spectrum and the calculated spectrum

obtained by using the parameters from RIXS results: the crystal field 10Dq = 1.13 eV for octahedral Fe^{2+} and Fe^{3+}, 10Dq = −0.6 eV for tetrahedral Fe^{3+}, and $\Delta_{t_{2g}} = -24$ meV. The calculated XAS agrees will the measured XAS after correction for self absorption.

Supplementary Note 3
Band structure calculations

We performed band structure calculations[5] for the low-temperature $P2/c$ structure of magnetite using the accurate frozen-core full potential projector augmented wave method, as implemented in the VASP package. The calculations are based on the generalized gradient approximation (GGA). Supplementary Figure 7 shows the calculated band structure to highlight the splitting of the t_{2g} bands at the Γ point due to the tetragonal distortion in the $P2/c$ structure. We found that the splitting between the d_{xy} and $d_{yz/zx}$ is 52 meV.

Supplementary Note 4
Single-crystal Synthesis and Characterisation

Single-crystal growth of magnetite was carried out in an infrared image furnace (NEC model SC-M35HD) in high-purity argon gas (99.999% purity) atmosphere at the Department of Mechanical Engineering, the University of Texas at Austin. The feed and seed rods, made of Fe_3O_4 powder (99.999% purity), were first loaded into a rubber tube and were compressed to 4 kbar of hydrostatic pressure. The compacted rods were then sintered in the furnace at 937°C with an O_2 gas flow for 20 hrs. The crystal was grown with the growing rate of 8 mm/h and the feed and seed rods being rotated in opposite directions with a rotation speed of 30 rpm each in the gas flow of high purity argon. Laue X-ray back diffraction patterns were used to orient the synthesised single crystal, which confirmed the cubic space group of Fd-3m with the lattice parameter a = 8.396 Å, see Supplementary Fig. 10. Measurements of the temperature-dependent specific heat and resistivity of the synthesized single-crystal magnetite showed that the Verwey transition occurred sharply with the first-order character at $T_V = 122$ K (Supplementary Fig. 11)[3;4]. It is well-known that the transition temperature T_V gets lowered and the transition becomes second order for Fe-deficient magnetite[3]. Based on these analyses, the synthesised single crystal has a chemical composition of $Fe_{3(1-\delta)}O_4$ with $|\delta| \leq 0.00018$, which indicates a nearly ideal chemical stoichiometry.

Supplementary References

[1] Lai, C. H. *et al.* Highly efficient beamline and spectrometer for inelastic soft X-ray scattering at high resolution. *J. Synchrotron Radiat.* **21**, 325-332 (2014).

[2] Stavitski, E. and de Groot, F. M. F. The CTM4XAS program for EELS and XAS spectral shape analysis of transition metal L edges. *Micron* **41**, 687-694 (2010).

[3] Shepherd, J. P., Koenitzer, J. W., Aragon, R., Spalek, J., Honig, J. M. Heat capacity and entropy of nonstoichiometric magnetite $Fe_{3(1-\delta)}O_4$: The thermodynamic nature of the Verwey transition. *Phys. Rev. B* **43** 8461 (1991).

[4] Zhou, J.-S., Goodenough, J.B., Dabrowski, B. Pressure-induced non-Fermi-liquid behavior of $PrNiO_3$. *Phys. Rev. Lett.* **94** 226602 (2005).

[5] Jeng, H. T., Guo, G. Y., & Huang, D. J. Charge-orbital ordering in low-temperature structures of magnetite: GGA+U investigations. *Phys. Rev. B* **74**, 195115 (2006).
